# Luteolin Inhibits Listeriolysin O Translation by Directly Targeting the Coding Region of the *hly* mRNA

**DOI:** 10.3389/fmicb.2019.01496

**Published:** 2019-07-02

**Authors:** Jianfeng Wang, Shui Liu, Bowen Liu, Xiaodi Niu, Xuming Deng

**Affiliations:** ^1^Department of Respiratory Medicine, The First Hospital of Jilin University, Changchun, China; ^2^Key Laboratory of Zoonosis Research, Ministry of Education, College of Veterinary Medicine, Jilin University, Changchun, China

**Keywords:** *Listeria monocytogenes*, listeriolysin O, luteolin, anti-infective therapy, *hly*

## Abstract

Listeriolysin O (LLO) is necessary for bacterial escape from the phagosome into the cytoplasm, which suggests that targeting LLO may be an alternative strategy to combat *Listeria monocytogenes*-mediated infection. Here, luteolin, a natural compound without anti-bacterial activity, as indentified as effective inhibitor of LLO by translationally inhibiting the production of LLO. Additionally, luteolin-treated *L. monocytogenes* displayed reductions in cytoplasmic growth, cytotoxicity and phagosome escape within macrophages. Molecular modeling and mutational analysis revealed a direct interaction between luteolin and the 5′ coding region (A818, U819, G820, and U830 located in nt 814–849) of the mRNA of *hly*, the gene encoding LLO, which interfered with its translation. Together, our data demonstrate that luteolin may be used as a novel therapeutic and lead compound for treating *L. monocytogenes* infection.

## Introduction

*Listeria monocytogenes*, a rare but deadly microbe, is an intracellular pathogen that is responsible for the disease listeriosis in humans and other animal species. This disease is usually caused by the ingestion of food contaminated with *L. monocytogenes*, which can grow well at refrigerated temperatures (4–10°C), as well as at low pH and high salt concentrations, all of which are typically used for food preservation (Dussurget et al., 2014). In recent years, an increasing incidence of listeriosis has been reported in the United States and several European countries, and the mortality rate associated with this infection has increased to approximately 30%, especially among immunocompromised individuals, even with early antibiotic treatment (Marini et al., 2016; Chen et al., 2017). The 2011 listeriosis outbreak in the United States was the worst foodborne illness-related incident in that country. Infections were reported in 28 states, and the outbreak lasted approximately 100 days. During this disaster, there were a total of 146 patients with invasive disease, 30 deaths and one miscarriage. Those who died ranged in age from 48 to 96 years, with a median age of 82.5 years (McCollum et al., 2013).

Molecular and cell biological approaches revealed that *L. monocytogenes* has evolved an elaborate arsenal of virulence factors (such as internalin, listeriolysin O, phospholipase C, LIPI III, LIPI IV, and ActA) that are expressed at the correct time and within the correct host environment to facilitate the pathogenicity of this organism during different stages of its intracellular life cycle (Cotter et al., 2008; Camejo et al., 2011; Maury et al., 2016; Chen et al., 2018). After internalization, acritical step in *L. monocytogenes* pathogenesis is the escape of the bacterium from the membrane-bound compartment (vacuole); failure to reach the cytosol results in bacterial degradation and death. Destabilization of the primary and secondary vacuolar membrane is largely mediated by listeriolysin O (LLO), a member of the cholesterol-dependent cytolysin family, suggesting that this toxin (encoded by the *hly* gene) is an essential virulence factor of *L. monocytogenes* (Hamon et al., 2012). Mutants lacking *hly* lose hemolytic activity, fail to escape from the vacuole, and display avirulence in a mouse model of infection. Moreover, as reported in a previous study by Edelson et al. (1999), a neutralizing mAb against LLO limits *L. monocytogenes* growth in infected cells and provides resistance against this bacterial infection in mice (Edelson and Unanue, 2001). Thus, instead of bacterial eradication, which mainly aims to disrupt cell wall biosynthesis, DNA replication and protein synthesis, targeting LLO may be a potential alternative strategy for treating *L. monocytogenes* infections.

**FIGURE 1:**
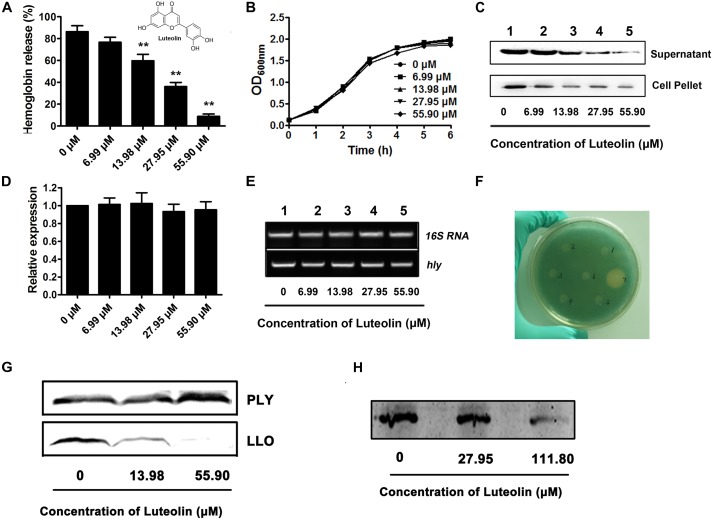
Luteolin inhibits LLO production at the translational level by directly targeting the coding region of *hly*. **(A)** The hemolytic activity in culture supernatants of *Listeria monocytogenes* following coculture with various concentrations of luteolin. **(B)** The growth of *L. monocytognes* in the presence of various concentration of luteolin. **(C)** Western blot analysis of LLO production in culture supernatants and bacterial pellets following luteolin treatment. **(D,E)** *hly* transcription in *L. monocytogenes* was not inhibited by luteolin. Bacteria were cocultured with various concentrations of luteolin, and the transcription of *hly* was evaluated by RT-PCR. **(F)** *L. monocytogenes-lacZ* fusions were cultured in TSB agar supplemented with X-gal and kanamycin at 37°C for 48 h to determine the effect of the tested compounds on *hly* transcription. Wells 1 to 5 contained 6.99, 13.98, 27.95, 55.90, and 111.80 μM luteolin, respectively; well 6 contained DMSO; and well 7 contained bromo-geramine. **(G)** The effect of luteolin on LLO and PLY production (transcription and translation) in a cell-free system. **(H)** The activity of luteolin in inhibiting LLO translation. *hly* mRNA was synthesized using a T7 RiboMAX Express Large-Scale RNA Production System and further used for the determination of LLO translation with an *E. coli* S30 Extract System for Linear Templates following luteolin treatment. The data are expressed as the mean ± SEM (*n* = 3). ^∗∗^*P* < 0.01.

Luteolin (Figure 1A) is a member of a group of naturally occurring compounds called flavonoids, which are commonly found in vegetables, fruits and natural herbal drugs, and has been proven to possess antioxidant, antihypertensive, anti-inflammatory, anticancer, and antiestrogenic activities (Lopez-Lazaro, 2009). Our previous studies also demonstrated that luteolin reduces alpha-hemolysin production by *Staphylococcus aureus* in a dose-dependent manner (Qiu et al., 2011). However, the effect of this compound against LLO has not been reported. Here, the influence of luteolin on the production of LLO and the growth of *L. monocytogenes* in infected cells was analyzed. Moreover, we assessed the potential mechanism of such influence.

## Materials and Methods

### Bacterial Strains, Growth Conditions, and Reagents

The *L. monocytogenes* strain EGDe (BUG 1600) and its LLO mutant, EGDe□ Δ*hly* (BUG 3649), were gifts from Dr. Pascale Cossart (Institut Pasteur, Paris, France). For hemolysis, Western blot and gene expression analyses, the *L. monocytogenes* strains were grown to the post-exponential phase [optical density at 600 nm (OD_600_) of 2.0] at 37°C in tryptic soy broth (TSB) with graded subinhibitory concentrations of luteolin. For the cell infection, the bacterial strains were cultured in TSB at 37°C to an OD_600_ of 0.8 (mid-exponential phase). Bacterial pellets from 10 ml of culture were washed with PBS and resuspended in cell culture medium or PBS to reach the indicated concentration.

Luteolin (purity ≥ 98%) was purchased from the National Institutes for Food and Drug Control (Beijing, China) and dissolved in dimethyl sulfoxide (DMSO) to make stock solutions of various concentrations.

### Antibacterial Activity Determination

*Listeria monocytogenes* was cultured in TSB at 37°C to an OD_600_ of 1.0 and diluted to a density of 5 × 10^5^ CFU/ml. An MIC test was performed using the broth microdilution method according to the procedures in the CLSI guidelines (M31-A2). In addition, the growth of *L. monocytogenes* treated with various concentrations of luteolin was monitored by determining the OD_600_ of each sample every 60 min.

### Hemolytic Activity Assay

Hemolytic activity analysis was performed as described previously with slight modifications (Qiu et al., 2011). Briefly, 100 μl of bacterial culture supernatant, 875 μl of hemolysis buffer (35 mM sodium phosphate, 125 mM sodium chloride, and 0.5 mg/ml BSA, pH 5.5 using acetic acid) and 25 μl of sheep erythrocytes were mixed and incubated at 37°C for 30 min. Following centrifugation (5500 × *g* for 1 min), the supernatants were used to examine the hemolytic activity by determining the optical density of each sample at 543 nm. Samples treated with 1% Triton X-100 served as 100% hemolysis controls, and the percentage of hemolysis was calculated by comparing each sample to the control culture.

### Western Blotting

To detect secreted LLO, 20 μl of bacterial culture supernatant was subjected to SDS-PAGE, and the protein was transferred to polyvinylidene fluoride (PVDF) membranes (Wako Pure Chemical Industries, Ltd., Osaka, Japan). The membranes were probed with a primary rabbit anti-LLO antibody and secondary horseradish peroxidase-conjugated anti-rabbit antiserum. Western blot detection was performed using Amersham ECL Western blotting detection reagents (GE Healthcare, Buckinghamshire, United Kingdom) and a chemiluminescence imaging system.

To detect surface-associated and internal LLO, bacterial pellets from 300 ml of culture were resuspended in 40 ml of PBS and adjusted to their final concentrations with 10 mM vanadate, 1 mM dithiothreitol (DTT), and 1× proteinase inhibitor mix (Roche Diagnostics), and the cells were then lysed by sonication. The lysates were subjected to SDS-PAGE and subsequent Western blotting, as described above.

### Real-Time Reverse Transcription-Polymerase Chain Reaction (RT-PCR)

Total RNA was isolated from broth-grown bacteria using standard phenol–chloroform extraction methods (as described by Mujahid et al., 2013). The collected RNA (1 μg) was treated with RNase-free DNase I (Qiagen, Hilden, Germany) to remove contaminating DNA and then subjected to reverse transcription using a Takara RNA PCR kit (AMV) ver 3.0 (Takara, Kyoto, Japan) according to the manufacturer’s instructions. PCR was performed in 25 μl of SYBR Premix Ex Taq^TM^ (Takara), as recommended by the manufacturer. All samples were analyzed in triplicate, and the relative expression of the *hly* gene was determined by comparing the transcript levels of these genes to those of bacterial *16S rRNA*, which was used as a reference gene. The DNA sequences of the PCR primers were as follows: *hly*, 5′-GAGCCTAACCTATCCAG-3′ (forward) and 5′-TGTAAGCCATTTCGTCA-3′ (reverse); and *16S RNA*, 5′-CTTCCGCAATGGACGAAAGT-3′ (forward) and 5′-ACGATCCGAAAACCTTCTTCATAC-3′ (reverse).

### Generation of *lacZ-*Fused *hly* and β-Galactosidase Assays

The pTCV-lac vector containing *lacZ* fused to *hly* was kindly gifted by Dr. Birgitte H. Kallipolitis (Department of Biochemistry and Molecular Biology, University of Southern Denmark) and was transformed into the EGDe□ Δ*hly* strain as described previously (Larsen et al., 2006). The bacteria containing promoter:*lacZ* fusions were grown in TSB and diluted 1000 times in PBS. The diluted bacteria (1 ml) were mixed in a Petri dish (9 cm diameter) with TSB agar supplemented with X-gal (150 μg/ml) and kanamycin (50 μg/ml). Luteolin (20 μl) at different concentrations was added to the wells, which were made using a sterilized drill. The plates were incubated at 37°C for 48 h. DMSO was used as a negative control, and bromo-geramine served as a positive control.

### *In vitro* Transcription and Translation Assay

The truncated *hly* gene was amplified from the pET21a^+^ LLO vector maintained in our laboratory and cloned into the pET21a^+^ vector with the BamH I and Xho I restriction enzyme sites. The oligonucleotide primers used in this study listed in Table 1. The *in vitro* production (transcription and translation) of LLO or truncated LLO in the presence of the indicated concentrations of luteolin was synthesized based on the vectors containing the *hly* gene or truncated *hly* with an S30 T7 High-Yield Protein Expression System according to the manufacturer’s protocols. The pET21a^+^ PLY vector (encoding pneumolysin, another member of the cholesterol-dependent cytolysin family) from our laboratory was used as an unrelated control. *hly* mRNA was produced using the T7 RiboMAX^TM^ Express Large-Scale RNA Production System based on the pET21a^+^ LLO vector, and the translation of *hly* mRNA was evaluated with an *E. coli* S30 Extract System for Linear Templates. The wild-type sequence (nt 814–849) and mutant sequences of *hly* were synthesized and cloned into the pET21a^+^ vector containing a luciferase reporter gene, and the recombinant luciferase was synthesized using the S30 T7 High-Yield Protein Expression System. The Renilla luciferase assay system was used to examine luciferase activity, as described previously (Larsen et al., 2006). Each recombinant plasmid was verified by double-strand DNA sequencing. The production of the protein of interest (PLY, LLO, truncated LLO, or luciferase) driven by the T7 promoter was determined using Western blotting, as described above, with an anti-His tag mouse monoclonal antibody and secondary horseradish peroxidase-conjugated anti-mouse antiserum.

**Table 1:** Primer sequences used for PCR.

Primer	Sequence (5′–3′)
1∼753bp-F	cgcGGATCCgatgcatctgcattcaat
1∼753bp-R	ccgCTCGAGtttagtaacagctttgccg
754∼1512bp-F	cgcGGATCCgagcagttgcaagcgcttg
754∼1512bp-R	ccgCTCGAGttcgattggattatctacttta
1∼564bp-F	cgcGGATCCgatgcatctgcattcaat
1∼564bp-R	ccgCTCGAGactgtaagccatttcgtcatc
565∼1011bp-F	cgcGGATCCgaatcacaattaattgcgaa
565∼1011bp-R	ccgCTCGAGgttgccgtcgatgatttgaac
1012∼1512bp-F	cgcGGATCCctcggagacttacgcgatat
1012∼1512bp-R	ccgCTCGAGttcgattggattatctacttta
799∼1512bp-F	cgcGGATCCtatatctcaagtgtggcgtat
799∼1512bp-R	ccgCTCGAGttcgattggattatctacttta
850∼1512bp-F	cgcGGATCCaattcccatagtactaaagta
850∼1512bp-R	ccgCTCGAGttcgattggattatctacttta
910∼1512bp-F	cgcGGATCCtcaggtgatgtagaactaaca
910∼1512bp-R	ccgCTCGAGttcgattggattatctacttta
961∼1512bp-F	cgcGGATCCgtaatttacggaggttccgca
961∼1512bp-R	ccgCTCGAGttcgattggattatctacttta
814∼1512bp-F	cgcGGATCCgcgtatggccgtcaagtttat
814∼1512bp-R	ccgCTCGAGttcgattggattatctacttta
832∼1512bp-F	cgcGGATCCtatttgaaattatcaactaat
832∼1512bp-R	ccgCTCGAGttcgattggattatctacttta
850∼1512bp-F	cgcGGATCCaattcccatagtactaaagta
850∼1512bp-R	ccgCTCGAGttcgattggattatctacttta


### Cell Culture and Infection

Mouse J774 macrophage-like cells were grown at 37°C and 5% CO_2_ in Dulbecco’s modified Eagle’s medium (DMEM; Invitrogen) with high glucose supplemented with 10% HI-FBS, 100 U/ml penicillin, and 100 μg/ml streptomycin. For intracellular proliferation, LDH release and confocal microscopy analyses, the cells were cultured and infected at bacterium:macrophage ratios of 5:1, 5:1, and 2.5:1, respectively.

### Intracellular Growth Assay

The J774 macrophage-like cells (5 × 10^5^ cells) were seeded onto 12 mm coverslips and incubated overnight in antibiotic-free medium. Bacteria (2.5 × 10^6^) suspended in medium with or without luteolin were cocultured with the J774 cells for 30 min, followed by three washes with prewarmed PBS. The culture medium was replaced with fresh medium containing 20 μg/ml gentamicin to kill the extracellular bacteria. One hour later, the gentamicin-containing medium was removed using three washes with prewarmed PBS and then changed to antibiotic-free tissue culture medium with or without luteolin for the remainder of the experiment. At the indicated time points, the number of bacteria associated with each coverslip was determined by placing the coverslip in sterile distilled H_2_O, vortexing vigorously for 30 s, and plating dilutions on TSB agar plates.

### LDH Release Assay

J774 macrophages were grown overnight in a 96-well plate at a density of 2 × 10^4^ cells per well prior to infection with 10 μl of bacterial suspension (1 × 10^7^ CFU/ml) with the indicated concentrations of luteolin. Five hours after infection, the supernatants from each well were collected for analysis of LDH activity using a Cytotoxicity Detection Kit (LDH; Roche, Basel, Switzerland) according to the manufacturer’s directions. This assay was performed in the absence of gentamicin to favor host cell lysis over rapid bacterial death and host cell repair. Relative LDH release of each sample = (OD of tested sample – OD of negative sample)/(OD of positive sample – OD of negative sample) × 100%.

### Processing for Confocal Microscopy

J774 macrophage-like cells were exposed to bacteria at a ratio of 2.5 bacteria per cell and processed for internalization and extracellular bacterial eradication as described for the intracellular growth assay. At 0.5, 2, or 5 h after infection, the cells were fixed with 4% (wt/vol) paraformaldehyde, and double fluorescence staining for F-actin and bacteria was performed using phalloidin coupled to Alexa 488 (Molecular Probes) and a rabbit antiserum for *L. monocytogenes* (Abcam, Cambridge, United Kingdom) tagged with an Alexa Fluor 594 chicken anti-rabbit IgG antibody (Molecular Probes). The survival of intercellular bacteria was also determined using a LIVE/DEAD^®^ BacLight^TM^ Bacterial Viability Kit at 5 hpi, as described previously (Wang et al., 2015). Microscopic images were captured using a laser-scanning confocal microscope (Olympus, Tokyo, Japan).

### Statistical Analysis

All experimental data are expressed as the mean ± SEM. Student’s *t*-test was used to calculate *P*-values using GraphPad Prism 5.0 software. Probabilities of 0.05 or less were considered statistically significant.

## Results

### Luteolin Translationally Inhibits LLO Production

A hemolysis assay was employed to screen potential active compounds against LLO production (Wang et al., 2015). The hemolytic activity in the culture supernatant of the EGDe *L. monocytogenes* strain was significantly attenuated after coculture with luteolin (Figure 1A). The concentrations required for this inhibition (13.98–55.90 μM) were much lower than the minimal inhibitory concentration (MIC) (447.20 μM) of luteolin for EGDe. Additionally, the bacterial growth was not visibly affected with luteolin treatment at the concentrations used in the hemolysis assay (Figure 1B). These results indicated that the luteolin-induced inhibition of supernatant hemolytic activity was not mediated by bacteriostatic or bactericidal activity. A Western blot assay was performed to further assess whether the decreased hemolytic activities were due to the inhibition of LLO secretion into the supernatant. Consistent with the results of the hemolysis assay, compared to the controls, luteolin treatment markedly reduced the secretion of LLO into the supernatant in a concentration-dependent manner (Figure 1C). Additionally, the LLO protein levels in lysed bacterial cells were examined to exclude the possibility that the reduced LLO protein levels in the supernatant were due to a blockade of luteolin-induced export. As expected, luteolin significantly suppressed LLO production in the cytoplasm in a dose-dependent manner (Figure 1C), which suggested that luteolin represses LLO protein synthesis and does not impede protein export.

The transcript level of the *hly* gene in *L. monocytogenes* was examined to determine whether luteolin could downregulate this gene. Interestingly, as measured by quantitative RT-PCR, the *hly* transcript level was similar in the cells treated with or without luteolin (Figure 1D,E). In addition, using an agar-based screening assay, the transcription of *hly* was evaluated using the EGDe Δ*hly* strain containing a promoter:*lacZ* fusion. Consistent with the results from a previous study (Kastbjerg et al., 2010), the addition of disinfectants (bromo-geramine) effectively attenuated the expression of *hly* (Figure 1F), while no inhibition of *hly* expression was observed in the presence of luteolin (Figure 1F). These results indicated that luteolin treatment had no influence on the transcription of *hly* and that the attenuation of LLO production by luteolin may be due to the inhibition of *hly* translation.

LLO production was measured via *in vitro* protein synthesis in a cell-free system in the presence of luteolin. As expected, the production of LLO was attenuated following luteolin treatment (Figure 1G). However, the addition of luteolin to the protein synthesis system caused no visible inhibition of PLY (Figure 1G), another member of the cholesterol-dependent cytolysin family that is produced by *Streptococcus pneumoniae*, suggesting that luteolin specifically inhibits LLO production. Furthermore, *hly* mRNA was generated using the T7 RiboMAX^TM^ Express Large-Scale RNA Production System, and the synthesized mRNA was used to evaluate the influence of luteolin on LLO production at the translational level. Consistent with our hypothesis, LLO production was significantly reduced following luteolin treatment in the protein translation system (Figure 1H). Together, our results show that luteolin, an agent without anti-*L. monocytogenes* activity, inhibits the translation of LLO.

### Luteolin Directly Interacts With the Coding Region of *hly* to Inhibit LLO Production

**FIGURE 2:**
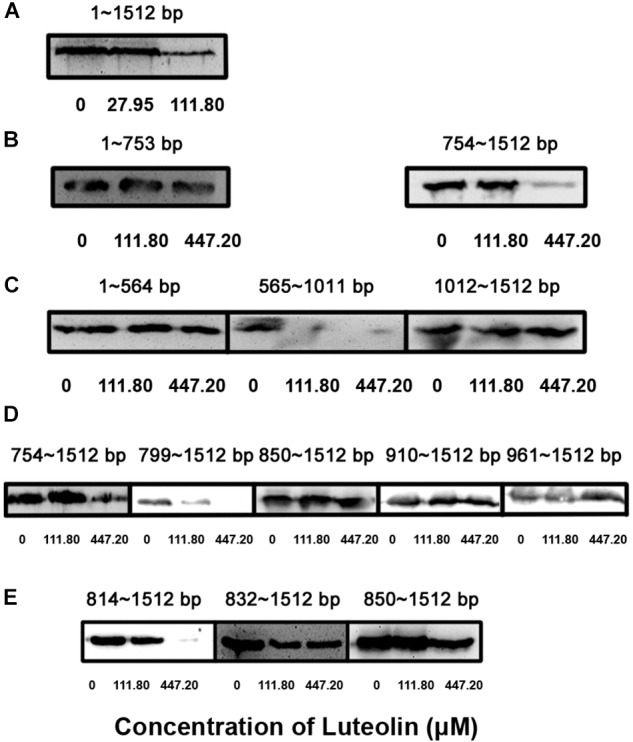
Identification of the key nucleotide sequence required for the inhibition of LLO production by luteolin. The *hly* gene was truncated and cloned into the pET21a^+^ vector. Truncated LLO was synthesized *in vitro* using a S30 T7 High-Yield Protein Expression System based on constructed expression plasmids in the presence of the indicated concentrations of luteolin. Then, the total production of truncated LLO in the protein expression system was determined using Western blotting. **(A–E)** The production of truncated LLO from the indicated nucleotide sequence.

The translational inhibition of LLO production by luteolin prompted us to determine the nucleotides in *hly* that are important for the interaction with this compound. Different *hly* gene truncations were cloned into the pET21a^+^ vector to screen for the region of *hly* involved in this inhibition. Consistent with the above results, luteolin treatment inhibited LLO production via the *hly* gene (bp 1–1512) (Figure 2A). Using the *in vitro* LLO synthesis system, the coding region of the *hly* gene (bp 754–1011) was identified as the target for luteolin interaction (Figure 2B,C). This region was further divided, and we found that the coding region from nt 814–849 was required for the luteolin-induced inhibition of LLO production (Figure 2D,E).

**FIGURE 3:**
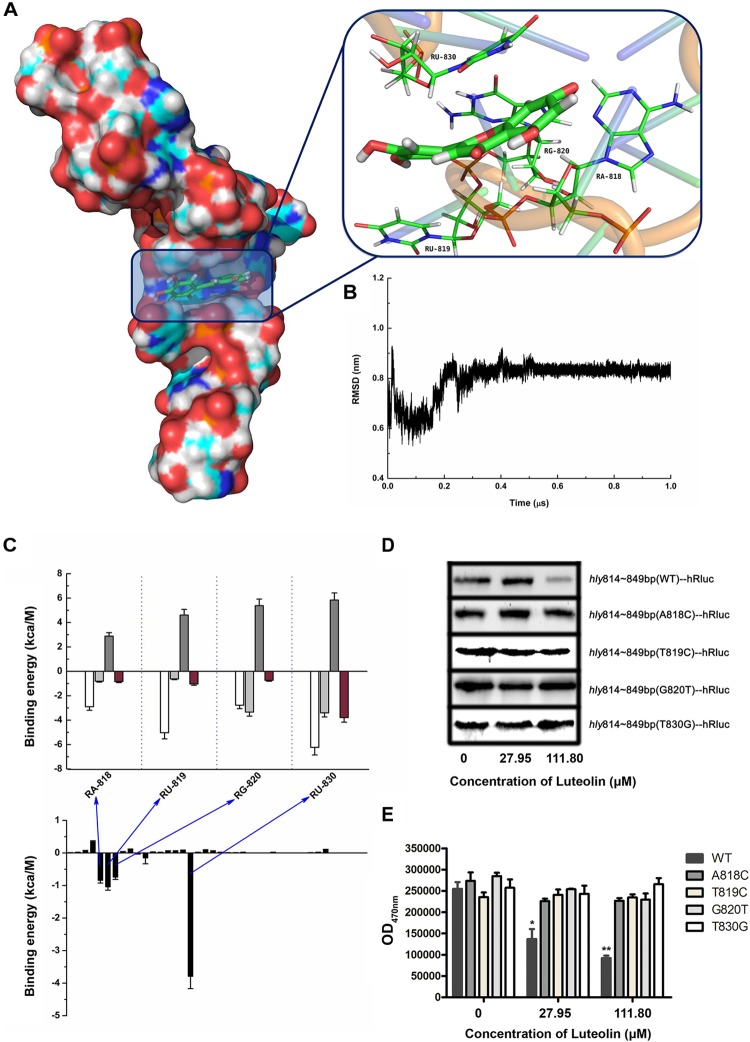
Molecular modeling of the binding mechanisms of luteolin to *hly* mRNA. **(A,B)** The binding mode of luteolin to mRNA based on MD simulation. The 3D structure and the bases in the binding site in the complex **(A)** and the RMSD of the backbone atoms of the mRNA with luteolin **(B)**. **(C)** Decomposition of the binding energy per base in the binding site of the mRNA-luteolin complex. A818, U819, G820, and U830 had the strongest interaction with luteolin. **(D)** The effect of luteolin on the production of recombinant luciferase with the *hly* sequence (nt 814–849) or a mutant version. The luciferase reporter gene with the wild-type (nt 814–849) or mutant *hly* sequence was cloned into pET21a^+^, and the recombinant protein was synthesized as described above. **(E)** The luciferase activity in the samples containing the wild-type (nt 814–849) or mutant *hly* sequence was measured using the Renilla luciferase assay system. The data are expressed as the mean ± SEM (*n* = 3). ^∗^*P* < 0.05 and ^∗∗^*P* < 0.01.

Molecular modeling was performed to further validate and identify the mechanism by which luteolin inhibits *hly* translation. As shown in Figure 3A,B, luteolin localized to the central bulged region of the mRNA during a 1 μs molecular dynamics (MD) simulation. The binding mode of mRNA-luteolin revealed strong interactions between luteolin and the A818, U819, G820, and U830 mRNAs (Figure 3A,B). To obtain detailed information about the bases in the binding region between luteolin and mRNA and their contribution to the whole system, the ligand-base interaction decomposition was calculated for the complex system using the MM-PBSA method. As shown in Figure 3C, A818, U819, G820, and U830 had the strongest interactions, with van der Waals values of -2.90, -5.03, -2.78, and -6.23 kcal/mol, respectively. Moreover, G820 and U830 also showed appreciable electrostatic contributions, with ΔEele values of -3.34 and -3.4 kcal/mol, respectively (Figure 3C). Except for G820, the majority of the decomposed energy interaction originated from van der Waals interactions, while electrostatic contributions appeared to have a minor influence on these key bases. Based on the above results, A818, U819, G820, and U830 may be important for the binding of luteolin (Figure 3C).

To confirm this hypothesis, wild-type or mutant sequences of *hly* nt 814–849 were cloned into the pET21a^+^ vector containing a luciferase reporter gene. As expected, the production of recombinant luciferase was reduced following luteolin treatment in the sample with the wild-type nucleotide sequence (nt 814–849) (Figure 3D). However, the recombinant luciferase production in the samples with mutant nucleotide sequences (A818C, T819C, G820T, and T830G) was not visibly affected by luteolin (Figure 3D). Consistent with these results, the inhibitory effect on Renilla luciferase activity in the samples with mutant nucleotide sequences (A818C, T819C, G820T, and T830G) was much weaker than that in the sample with the wild-type nucleotide sequence (nt 814–849) (Figure 3E). Taken together, these results demonstrate that the interaction of luteolin with the coding region of the *hly* mRNA (A818, T819, G820, and T830) significantly inhibits the translation of LLO.

### Luteolin Blocks *L. monocytogenes* Phagosome Escape

**FIGURE 4:**
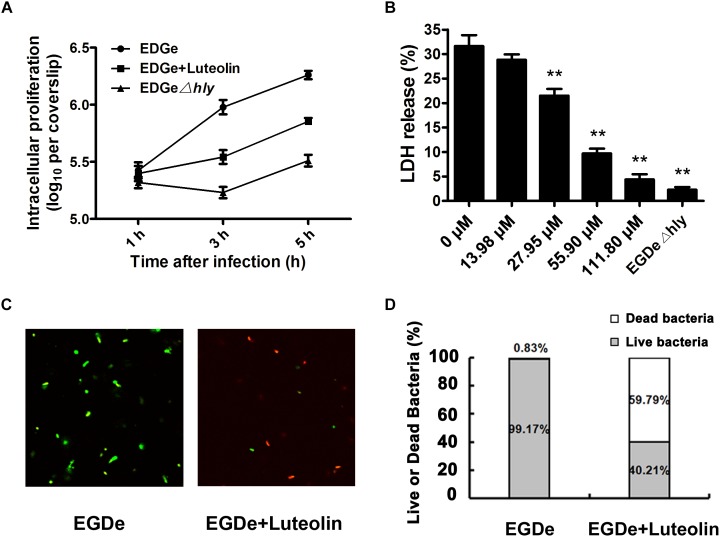
Luteolin impairs intracellular *L. monocytogenes* growth and cytotoxicity and facilitates bacterial degradation by macrophages. Mid-exponential phase bacteria were used to infect macrophages. At the indicated time points, the macrophages were lysed to count intracellular CFUs **(A)**. **(B)** After 5 h of infection, LDH release from the macrophages was assessed using a Cytotoxicity Detection Kit (LDH). Percentage cytotoxicity = 100 × (experimental LDH release - spontaneous LDH release)/(maximal LDH release – spontaneous LDH release). **(C,D)** At 5 hpi, lysates of infected macrophages were incubated with LIVE/DEAD^®^ BacLight^TM^ Bacterial Viability Kit reagents, which stain live (green) and dead bacteria (red). LDH, lactate dehydrogenase. The data are expressed as the mean ± SEM (*n* = 3). ^∗^*P* < 0.05 and ^∗∗^*P* < 0.01.

The observed inhibition of LLO production by subinhibitory concentrations of luteolin prompted us to examine the effect of this compound on cell infection by *L. monocytogenes*. The intracellular growth of bacteria in infected macrophages treated with or without luteolin was examined. EGDe Δ*hly* (in which LLO is deleted) was defective in intracellular replication in J774 macrophage-like cells (Figure 4A), which was consistent with previous reports and suggested that in macrophages, the intracellular growth of *L. monocytogenes* depends on the expression of LLO. However, as shown in Figure 4A, bacteria grown normally in host cells and exposed to 55.90 μM luteolin showed defective intracellular growth in J774 cells compared to control bacteria grown without luteolin.

We further monitored the release of a host cytosolic enzyme, LDH, into the culture medium of infected J774 cells to determine whether luteolin reduces *L. monocytogenes*-mediated host cell cytotoxicity. At 5 h post-infection (hpi), J774 cells infected with *L. monocytogenes* showed 31.63% of the maximal LDH release (Figure 4B), whereas 28.82, 21.52, 9.70, and 4.39% of the maximal LDH release was observed in cells treated with 13.98, 27.95, 55.90, and 111.80 μM luteolin, respectively (Figure 4B). Consistent with the intracellular growth analysis, LDH release of less than 2.5% was observed in macrophages infected with the EGDe Δ*hly* LLO-negative strain (Figure 4B).

**FIGURE 5:**
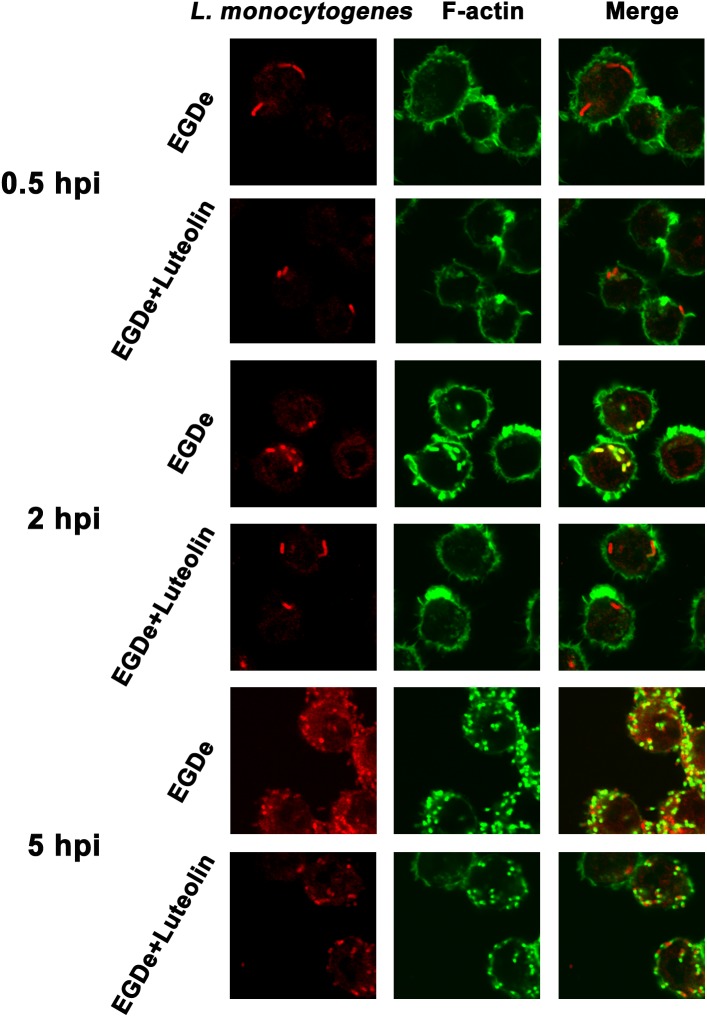
Luteolin inhibits *L. monocytogenes* phagosome escape and subsequent cytoplasmic growth in infected macrophages. Mid-exponential phase bacteria were used to infect macrophages. At the indicated time points post-infection, the cells were double stained with phalloidin coupled to Alexa 488 (actin – green) and a secondary chicken anti-rabbit IgG antibody conjugated to Alexa Fluor 594 (for all *L. monocytogenes* strains – red). Bacteria associated with F-actin sheaths are cytosolic. Imaging using the phalloidin channel was performed to identify and confirm the perimeters of the macrophages. **(A)** At 0.5 hpi, there was no visible difference in the number of bacteria in samples treated with or without luteolin, indicating that luteolin treatment did not affect the invasion of *L. monocytogenes*. **(B)** Most bacteria were surrounded by F-actin at 2 hpi, and almost all of the bacteria lacked F-actin sheaths in the sample treated with 111.80 μM luteolin, suggesting that the addition of luteolin blocks the escape of *L. monocytogenes* from the phagosome. **(C)** After 5 h of infection, robust replication was observed for the sample without luteolin treatment; many fewer bacteria and less actin polymerization were visible in infected cells treated with 111.80 μM luteolin.

Confocal microscopy was employed to directly evaluate the effects of luteolin on bacterial invasion, phagosome escape, and intracellular survival using double staining with an anti-*Listeria* antibody and phalloidin to visualize F-actin. At 0.5 hpi, no visible difference in bacterial invasion was observed between samples treated with or without luteolin (Figure 5), suggesting that luteolin treatment had no influence on bacterial invasion. Following invasion, bacterial phagosome escape was determined at 2 hpi. Consistent with previous results, intracellular bacteria successfully escaped from the phagosomes, as most bacteria were surrounded by polymerized actin in the macrophage cytosol (Figure 5). However, in the sample treated with luteolin, most bacteria were not stained with phalloidin (Figure 5). The EGDe *L. monocytogenes* strain had multiplied significantly at 5 hpi, and phagosome escape was evident (Figure 5). In particular, bacteria were scarce in infected J744 cells exposed to 55.90 μM luteolin, with actin comets rarely visible (Figure 5). The fate of *L. monocytogenes* in the host cells treated with or without luteolin was further examined by live/dead staining of the bacteria. In the sample without luteolin, almost all bacteria survived (Figure 4C,D). In contrast, 59.79% of the bacteria died in the sample treated with luteolin (Figure 4C,D), suggesting that luteolin treatment facilitated bacterial degradation by macrophages. Taken together, these results indicate that luteolin treatment inhibits intracellular bacterial phagosome escape, cytoplasmic replication and cytotoxicity, resulting in bacterial degradation by host cells.

## Discussion

Similar to *L. monocytogenes*, for most bacteria, pathogenicity is largely dependent on various virulence factors, such as pore-forming toxin, sortase, and secretion systems (Lee and Kessler, 2009; Camejo et al., 2011; Bradshaw et al., 2015). Therefore, targeting virulence factors may represent an alternative strategy for fighting bacterial infection. Additionally, compared to antibiotics, this strategy may put less selective pressure on the targeted bacteria (Cegelski et al., 2008; Rasko and Sperandio, 2010). Using hemolysis assays and Western blot analyses, we found that the natural compound luteolin is a promising antivirulence agent with the potential to inhibit the production of LLO, which has been recognized as one of the most important virulence factors in *L. monocytogenes*. In contrast to our previous studies (Qiu et al., 2011; Wang et al., 2011), luteolin treatment had no influence on the transcription of *hly*, the gene encoding LLO. However, the addition of luteolin to a cell-free protein system inhibited LLO production by directly targeting the coding region of *hly* at the translational level *in vitro*. Additionally, luteolin treatment specifically inhibited LLO production in *L. monocytogenes* and the cell-free protein system without affecting bacterial growth. Consistent with an earlier study that used an LLO inhibitor (Wang et al., 2015), luteolin-treated *L. monocytogenes* showed a marked reduction in phagosome escape and intracellular growth in macrophages host cells, which led to the robust degradation of drug-treated bacteria by macrophages. In addition, no therapeutic effect of luteolin against *Salmonella typhimurium* infection was reported previously (Tsuyoshi et al., 2013), suggesting that luteolin inhibits *L. monocytogenes* virulence by specifically targeting LLO. Targeting LLO using luteolin may be an alternative strategy for combating *L. monocytogenes* infection without selective pressure that fosters drug resistance.

Effective antivirulence inhibitors have been screened and identified by their ability to directly neutralize the biological activity of virulence factors or inhibit their production at the transcriptional level (Edelson et al., 1999; Edelson and Unanue, 2001; Qiu et al., 2012; Li et al., 2016). LLO plays an important role in the virulence of *L. monocytogenes*, as evidenced by the fact that *hly* gene knockout causes avirulence and gene complementation restores the pathogenicity of the mutant (Mengaud et al., 1989; Schnupf and Portnoy, 2007; Seveau, 2014). Studies focusing on the identification of LLO inhibitory agents (which inhibit the production or biological activity of LLO) are ongoing. Subinhibitory concentrations of β-lactams have been shown to repress the production of LLO (Nichterlein et al., 1996). Furthermore, previous findings have proven that the hemolytic activity of cholesterol-dependent cytolysins can be directly inhibited by epigallocatechin gallate and fisetin (Wang et al., 2015; Song et al., 2017). However, luteolin inhibited LLO production by directly interacting with the 5′ coding region (nt 814–849) of the *hly* mRNA in this study, suggesting that this compound is a potential inhibitor of LLO production via RNA. Numerous mRNA inhibitors that selectively inhibit the translation of a single mRNA transcript within the transcriptome have been characterized. Examples of such inhibition based on interaction with the 5′-UTR or 3′-UTR, whether mediated by antisense RNA, proteins, or small molecules, demonstrate that these targeting ligands can inhibit translation (Tor, 2003). However, inhibiting translation by directly targeting the coding region is relatively rare and is present frequently only in disease states (Tor, 2003). To further elucidate the potential molecular mechanism induced by luteolin, a standard MD simulation of the mRNA-luteolin complex was performed. Based on the MD simulation and a calculation of the binding free energy, we found that luteolin could bind to the central bulged region in the *hly* mRNA via strong interactions with A818, U819, G820 and U830, and these interactions were confirmed by ligand-residue interaction decomposition using the MM-PBSA method, point mutations of the bases, and a fluorescence-quenching assay. The interaction of luteolin with the *hly* mRNA restricted the inhibition of LLO translation. However, luteolin must enter the host cells and gain access to the intracellular compartment containing the bacteria to achieve such inhibition, which may hinder the use of luteolin as an LLO inhibitor for *L. monocytogenes* infection. To the best of our knowledge, this is the first example of an inhibitor of LLO production that is derived from a natural product and directly targets the coding region of the LLO-encoding gene.

Overall, our results raise the possibility of screening for or designing mRNA inhibitors via the novel strategy of directly targeting the coding region. Luteolin-induced inhibition of LLO production may also provide a paradigm for characterizing the molecular mechanism of mRNA inhibitors with similar modes of action and for developing anti-infection agents that specifically target bacterial virulence factors.

## Data Availability

The raw data supporting the conclusions of this manuscript will be made available by the authors, without undue reservation, to any qualified researcher.

## Author Contributions

XD, XN, and JW conceived and designed the experiments. JW, SL, and BL conducted the experiments. JW and SL contributed the reagents, materials, and analysis tools. JW and XD wrote the manuscript.

## Conflict of Interest Statement

The authors declare that the research was conducted in the absence of any commercial or financial relationships that could be construed as a potential conflict of interest.
